# Metacommunity patterns of Amazonian Odonata: the role of environmental gradients and major rivers

**DOI:** 10.7717/peerj.6472

**Published:** 2019-05-06

**Authors:** Fernanda Alves-Martins, Leandro Schlemmer Brasil, Leandro Juen, Paulo De Marco Jr, Juliana Stropp, Joaquín Hortal

**Affiliations:** 1 Departament of Biogeography and Global Change, Museo Nacional de Ciencias Naturales (MNCN-CSIC), Madrid, Madrid, Spain; 2 Instituto de Ciências Biológicas, Universidade Federal do Pará, Belém, Pará, Brazil; 3 Departamento de Ecologia, Instituto de Ciências Biológicas, Universidade Federal de Goiás, Goiânia, Goiás, Brazil; 4 Instituto de Ciências Biológicas e Saúde, Universidade Federal de Alagoas, Maceió, Alagoas, Brazil

**Keywords:** Amazonia biogeographical regions, Zygoptera, Anisoptera, Clementsian distributions, Habitat integrity, Macroclimate

## Abstract

**Background:**

We identified and classified damselfly (Zygoptera) and dragonfly (Anisoptera) metacommunities in Brazilian Amazonia, relating species distribution patterns to known biological gradients and biogeographical history. We expected a random distribution of both Zygoptera and Anisoptera within interfluves. At the Amazonian scale, we expected Anisoptera metacommunities to be randomly distributed due to their higher dispersal ability and large environmental tolerance. In contrast, we expected Zygoptera communities to exhibit a Clementsian pattern, limited by the large Amazonia rivers due to their low dispersal ability.

**Methods:**

We used a dataset of 58 first-to-third order well-sampled streamlets in four Amazonian interfluves and applied an extension of the Elements of Metacommunity Structure (EMS) framework, in which we order Zygoptera and Anisoptera metacommunities by known spatial and biogeographic predictors.

**Results:**

At the Amazonian scale, both Zygoptera and Anisoptera presented a Clementsian pattern, driven by the same environmental and biogeographical predictors, namely biogeographic region (interfluve), annual mean temperature, habitat integrity and annual precipitation. At the interfluve scale, results were less consistent and only partially support our hypothesis. Zygoptera metacommunities at Guiana and Anisoptera metacommunities at Tapajós were classified as random, suggesting that neutral processes gain importance at smaller spatial scales.

**Discussion:**

Our findings were consistent with previous studies showing that environmental gradients and major rivers limit the distribution of Odonata communities, supporting that larger Amazonian rivers act as barriers for the dispersal of this group. In addition, the importance of habitat integrity indicates that intactness of riparian vegetation is an important filter shaping metacommunity structure of Amazonian stream Odonata.

## Introduction

The greatest biodiversity, the world’s largest river system and tropical rainforest are located in the Amazonian region (Wesselingh et al., 2011). Not surprisingly, rivers have been at the focus of the hypotheses explaining how biodiversity is distributed across Amazonia. One of the oldest, yet widely accepted, of such hypotheses proposes that major rivers constrain species distributions (Wallace, 1852; Cracraft, 1985). Indeed, a distinct set of species is often observed on either side of a major river bank. This is particularly evident for taxa with low-dispersal ability such as monkeys (Wallace, 1852; Lynch Alfaro et al., 2015), frogs (Gascon et al., 2000; Godinho & Da Silva, 2018), and a few groups of birds (Bates, Haffer & Grismer, 2004). However, major rivers seem to be unimportant for shaping the distribution of taxa with high dispersal abilities. Plants (e.g., Tuomisto, 2003), butterflies (e.g., Penz et al., 2015), termites and likely other insects (e.g., Dambros et al., 2017) seem to have a more uniform community composition across river banks. For these taxa, the turnover in species composition may result from species’ response to changes in environmental conditions, both over long and short geographical distances (Tuomisto, Zuquim & Cárdenas, 2014), rather than their inability to cross a physical barrier.

The high variation in species composition on Amazonia may be also a result of environmental gradients at larger spatial scales, as the biome is highly heterogeneous in terms of climate and landscapes (Bush & Lovejoy, 2007). The Amazonian region encompasses diverse types of climate, soil and landscapes (Hoorn & Wesselingh, 2011). There are three distinct climatic zones in the region: a wet tropical climate without any dry season towards the west, a drier climate with a marked dry season during the austral winter towards the east, and a South America monsoon climate in the central portion of Amazonia (Alvares et al., 2013). Soils range from heavily leached (Steege et al., 2006; Quesada et al., 2009) and poorly drained sandy soils towards the North to clayey and more nutrient rich soils towards the West (Quesada et al., 2011). These changes in environmental conditions over large geographical distances has been consistently reported as an important predictor of turnover in species composition for ferns (Zuquim et al., 2012), trees (Steege et al., 2006; Quesada et al., 2009), ants (Vasconcelos et al., 2010), bees (Abrahamczyk et al., 2011), damselfies (Brasil et al., 2018), among others organism.

The Amazonian region, together with the Asian tropical forests, host the highest Odonata diversity in the world (Kalkman et al., 2008). Within this group, damselflies (suborder Zygoptera) and dragonflies (suborder Anisoptera) present distinct characteristics that result in different geographical patterns of diversity. Major Amazonian rivers have acted historically as dispersal barriers for the low-dispersal Zygoptera, delimiting centers of endemism and generating a large number of diversity hotspots. Many Zygoptera species have small ranges and are forest specialists (Paulson, 2004; Paulson, 2006; Kalkman et al., 2008; De Marco, Batista & Cabette, 2015). These characteristics are expected to create non-random distributional patterns, compared to the high-dispersal Anisoptera (Juen & De Marco, 2012). These differences outstand beyond the strong association of the distribution of all Odonata with both climate (Keil, Simova & Hawkins, 2008; Hassall et al., 2014; Bush et al., 2014) and the physical structure of habitats (e.g., Carvalho et al., 2013; Monteiro Jr, Juen & Hamada, 2015; De Marco, Batista & Cabette, 2015).

In this study, we aim to identify the role of physical barriers to dispersal (i.e., major rivers), environmental gradients and geographical distance as determinants of the spatial structure of Odonata metacommunities in the Brazilian Amazonia. More specifically, we investigate how the structure of the metacommunities varies within interfluves of major rivers and across the entire Amazonia basin, for Zygoptera and Anisoptera –two Odonata suborders with different dispersal abilities. Given that the probability of continuous flows of individuals increases at smaller spatial extents (Nekola & White, 1999), we expect a random distribution (i.e., lack of metacommunity structures) for both Zygoptera and Anisoptera within each of the interfluves studied. Further, we expect that the different dispersal abilities of both suborders combined with their responses to environmental gradients will result in different metacommunity patterns at larger extents (Bried et al., 2016). Therefore, we expect that the lower dispersal ability of Zygoptera will create a non-random metacommunity structure. Indeed, Zygoptera presents generally high turnover of species at large spatial extents (Bried et al., 2016; Brasil et al., 2018; Bried & Siepielski, 2018), following a Clementsian pattern (i.e., the response to environmental gradients produces discrete communities that replace each other; Leibold & Mikkelson, 2002) at least in in Amazonian non-impacted streams (Brasil et al., 2017). In contrast, the broader ecological amplitude (Remsburg & Turner, 2009; Mendes, Cabette & Juen, 2015; Soares et al., 2015) and higher dispersal ability of Anisoptera (Angelibert & Giani, 2003; McCauley, 2006; May, 2013) will result in a random metacommunity distribution across interfluves. If this expectation holds true, many Zygoptera species would have their distribution range limited by major rivers, while most Anisoptera will not present such limitation.

## Materials and Methods

### Sampling

We obtained standardized counts of individuals of Zygoptera and Anisoptera from the database presented by Juen & De Marco (2012). In their study, they sampled adult individuals of Zygoptera and Anisoptera in standardized surveys established in eight Amazonian biogeographic regions, that are confined by major rivers: Guiana (interfluve between Amazonas and Rio Negro), Imeri (interfluve between Rio Negro and Solimões), Napo (interfluve between Solimões and Napo), Inambari (interfluve between Solimões and Madeira), Rondônia (interfluve between Madeira and Tapajós), Tapajós (interfluve between Tapajós and Xingú), Xingú (interfluve between Xingú and Tocantins), and Belém (interfluve between Tocantins and Amazonas). Moreover, Juen & De Marco (2012) complemented their transect data with occurrences retrieved from the literature.

**Figure 1:**
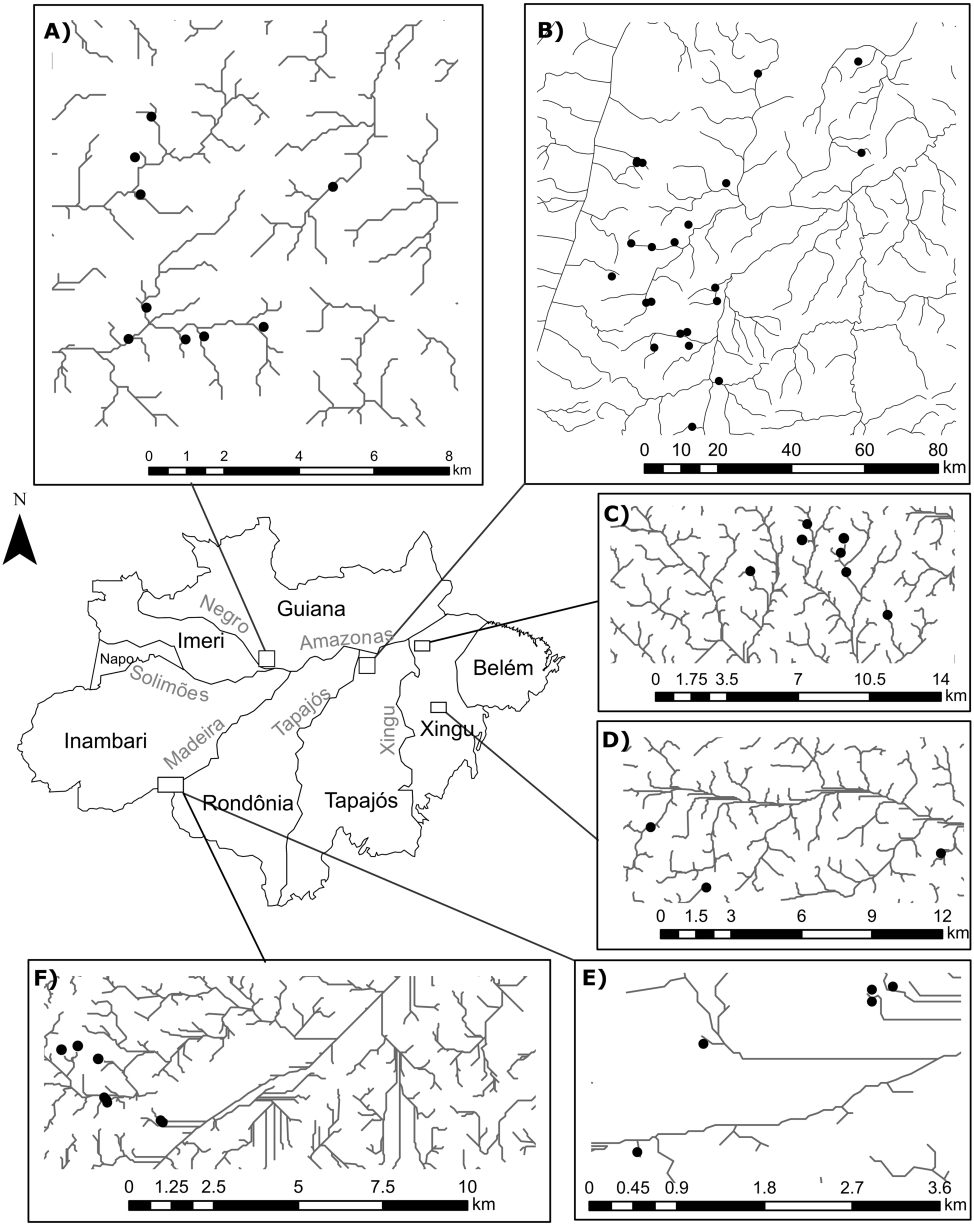
Geographical location of the surveyed streamlets in four interfluves of the Amazon basin: (A) Streamlets surveyed in the Guiana interfluve –Anisoptera (0), Zygoptera (9), Total (9); (B) Streamlets surveyed in the Tapajós interfluve –Anisoptera (6), Zygoptera (16); Total (21); (C and D) Streamlets surveyed in the Xingú interfluve –Anisoptera (4), Zygoptera (10); Total (10); (E and F) Streamlets surveyed in the Inambari interfluve –Anisoptera (5), Zygoptera (12); Total (12).

The number of sites in our study differs from Juen & De Marco (2012) because we excluded a few occurrences in order to avoid statistical problems arising from uneven sampling effort. To this end, we selected only occurrences that: (1) were collected during standardized surveys; (2) were collected in streamlets that showed an habitat integrity index (HII; Nessimian et al., 2008) greater than 0.7, thereby reducing the influence of anthropogenic impact in species composition (Brasil et al., 2018); and (3) belonged to surveys that recorded at least 15 individuals from each suborder. Moreover, we only included in our analyses streamlets with sample completeness (Chao & Jost, 2012) greater than 0.75. After this selection, we obtained 1673 Zygoptera specimens and 618 Anisoptera specimens collected in 58 first-to-third order streamlets, following (Strahler, 1957), in four Amazonian interfluves: Guiana (Anisoptera=0, Zygoptera=9, Total=9), Tapajós (Anisoptera=6, Zygoptera=16; Total=21); Xingú (Anisoptera=4, Zygoptera=10; Total=10); Inambari (Anisoptera=5, Zygoptera=12, Total=12) (Fig. 1). All the streams are located within areas of dense *terra-firme* rainforest that are typical of the Amazon biome (Myster, 2016).

Adult odonates were collected in the dry season (July–November) between 2009 and 2013, during peak of activity of odonates between 11h30 and 14h00 (Corbet, 1980). The greatest diversity of aquatic insects in the Amazon region is expected during this season (Baptista et al., 2001), and sampling is least likely to be affected by climatic conditions (Brasil et al., 2018). The basic sampling unit was defined as a 100 m long transect divided in 20 segments of 5 m that covered a segment of a streamlet. All adult odonates present in each segment were recorded in short scans with a sampling effort of 1.66 meter/min on average (De Marco, 1998; Brasil et al., 2014). This short time scan sampling protocol is broadly used (e.g., Juen, Cabette & De Marco, 2007; De Marco et al., 2014; Oliveira-Junior et al., 2015), for it prevents counting the same individual twice and guarantees that species with low population density are recorded. Specimens were collected with an entomological net and carried to the laboratory whenever required for species identification. The specimens were prepared following the protocol described by Lencioni (2005a) and Lencioni (2005b) were identified using taxonomic keys (e.g., Lencioni, 2005a; Lencioni, 2005b; Heckman, 2006), as well as comparing with voucher specimens available in the entomological collection of Zoological Museum of the Universidade Federal do Pará, Belém, Brazil.

**Figure 2:**
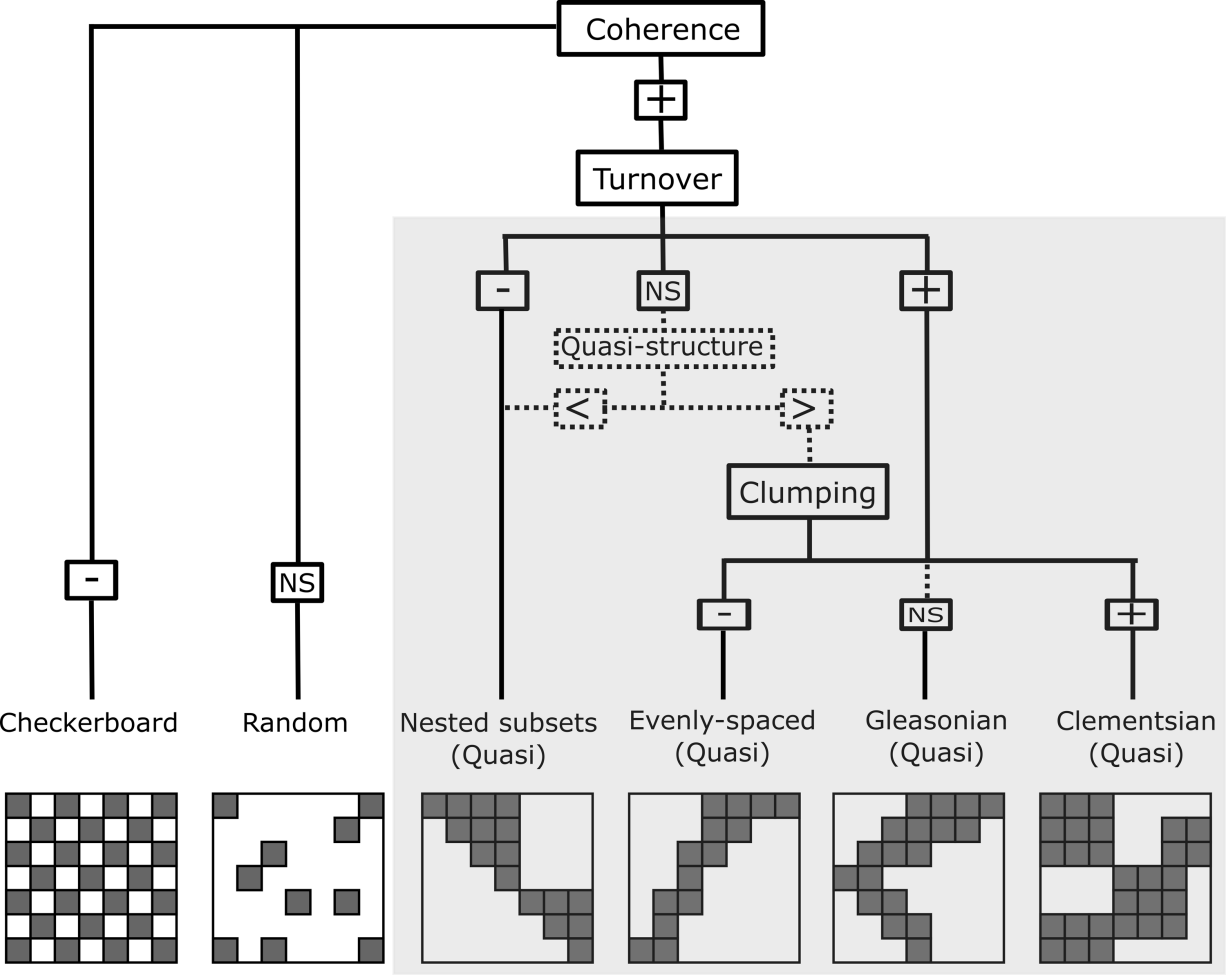
Representation of the six resulting metacommunity patterns and their structures and quasi-structures (in shaded area) generated from the combination of the three Elements of Metacommunity Structure. “+” means positive significance; “NS” means non-significance; (-) negative significance. The Quasi-structures are generated when turnover are not significant but exhibit trends. (<) indicates that the observed number of replacements is lower than the average number based on randomizations; (>) indicates that the observed number of replacements is higher than the average number based on randomizations. Adapted from Willig et al., (2011).

### Metacommunity structure analysis

In order to characterize the spatial structure on Amazonian Odonata metacommunities, we used an extension of the Elements of Metacommunity Structure (EMS) framework, proposed by Leibold & Mikkelson (2002) and modified by Presley, Higgins & Willig (2010) and Dallas et al. (2016). The EMS framework allows identifying six distinct idealized spatial structures that are outcomes of variation in species composition among communities: nested, Clementsian, Gleasonian, evenly spaced, random and checkerboard patterns (Fig. 2). The first step of the metacommunity structure analysis consists in ordering site-by-species incidence matrices. To this end, we rearranged the incidence matrix through reciprocal averaging (Correspondence analysis –CA), in such a way that species and sites are ordered along ordination axes based on species composition, which are defined as ‘latent environment gradients’ (Leibold & Mikkelson, 2002; Heino et al., 2015). Additionally, with the purpose of arranging Odonata communities (i.e., the “sites” of the matrix) by observed environmental gradients, we sorted sites by the following climatic variables: Annual Mean Temperature, Temperature Seasonality and Annual Precipitation, which were extracted from the WorldClim database (Fick & Hijmans, 2017) at a 10 min resolution. Moreover, we sorted sites by vegetation structure. For this, we used the MODIS enhanced vegetation index (EVI) that indicates the structural complexity of the vegetation around the sampling localities (Zellweger et al., 2015) and four additional habitat variables, which were measured in the field: stream depth, stream width, stream pH, and habitat integrity. We measured stream channel width because it is recognized to affect odonate composition at local sites (Juen & De Marco, 2011; De Marco, Batista & Cabette, 2015). Habitat Integrity Index (HII; Nessimian et al., 2008) measures the physical integrity of streamlets through the quantification of habitat attributes, such as completeness of riparian forest, bank structure or stream bottom, among others. We used HII as a proxy for intactness of riparian forest and the availability of microhabitats (Nessimian et al., 2008; Oliveira-Junior et al., 2017). We measured pH because it is a key factor affecting the composition of aquatic insects, including odonate larvae (Frolich Strong & Robinson, 2004; Couceiro et al., 2007; Al-Shami et al., 2013; Heino & Grönroos, 2013; Mendes et al., 2018). Other spatial gradients related to geographic distance were represented by the spatial filters (i.e axes) obtained by a Principal Coordinates of Neighbour Matrices of the geographical coordinates (PCNM: Dray, Legendre & Peres-Neto, 2006). Finally, we ordered sites by interfluves, as a proxy for the effects of biogeographical history and physical barriers to dispersal (except for the analysis within interfluve).

The second step of the structure analysis identifies which of the six idealized spatial structures (Fig. 2) are displayed by the metacommunities. To this end, the number of uninterrupted presences in the ordered matrix (i.e., coherence) is calculated for each of eleven ordered incidence matrices described above (Leibold & Mikkelson, 2002). Coherence reveals whether species are: (1) grouped because they are possibly following the same environmental gradient (Henriques-Silva et al., 2013) (i.e., positive coherence, which leads to one of the four patterns: nested, evenly spaced, *Gleasonian*, or *Clementsian*); (2) mutually exclude possibility due to competitive exclusion or evolutionary constraints (i.e., negative coherence, which leads to a checkerboard pattern); or (3) are randomly distributed (i.e., a random pattern).

The third step of the analysis determines whether species either are nested or replace each other along a spatial or environmental gradient. This was calculated by counting the number of species replacements for each pair of sites and the number of site replacements for each pair of species (see Almeida-Neto et al., 2008 for the rationale of assessing nestedness of both species and sites). When the number of replacements is significantly higher than the mean of replacements obtained by the randomized matrices, the metacommunity structure presents a positive turnover, thereby indicating that the majority of species in the metacommunity replace each other over a gradient. Positive turnover may be associated to Clementsian, Gleasonian or evenly spaced patterns, depending on the degree to which the distribution ranges of different species are clustered together (Presley, Higgins & Willig, 2010). Here, a *Clementsian* pattern is observed when species replace each other and their distribution ranges are clumped along a gradient (i.e., positive boundary clumping). Whereas a *Gleasonian* pattern is observed when there is species replacement, but the actual arrangement of their distribution ranges is random along the gradient. Lastly, an evenly spaced pattern emerges when distribution ranges are distributed more evenly than expected by chance. However, if instead of being replaced, species undergo a progressive loss across communities (i.e., negative turnover), a nested pattern emerge (Leibold & Mikkelson, 2002). Clumping of species distribution ranges was assessed by using the Morisita’s index (I) (Morisita, 1971) and a *χ*^2^ test.

Finally, the statistical significance of coherence and turnover were assessed using a null model based on permutation tests. Random matrices were produced with the algorithm ‘curveball’, which keeps fixed both species richness of a site and species frequencies (i.e row and column totals) (Strona et al., 2014). This algorithm is computationally fast, insensitive to the manner matrices are filled and configurated, creating truly null matrices (Strona et al., 2014; Bried & Siepielski, 2018). We performed statistical analyses using 999 iterations, in the “metacom” package (Dallas, 2014) in a R statistical coding environment (R Core Team, 2017). Here it is important to note that for Anisoptera only the Tapajós interfluve presented a minimum sample size for the analysis at the interfluve scale (*N* = 6 sites).

## Results

Our data comprises 122 Odonata species, with 69 species to the Zygoptera suborder and 53 species belonging to the Anisoptera suborder. The most frequently observed Zygoptera were *Mnesarete aenea* Selys, 1853 (Calopterygidae, 14.50%), *Argia thespis* Hagen in Selys, 1865 (Coenagrionidae, 7.24%), and *Chalcopteryx rutilans* Rambur, 1842 (Polythoridae, 6.65%). The most frequent Anisoptera were *Perithemis lais* Perty, 1834 (Libellulidae, 15.53%), *Erythrodiplax fusca* Rambur, 1842 (Libellulidae, 14.40%), *Erythrodiplax basalis* Kirby, 1897 (Libellulidae, 13.10%).

Consistent to our initial expectation, both Zygoptera and Anisoptera metacommunities displayed a random spatial structure within the interfluves of Guianas and Tapajós, respectively (Table 1). When we order Zygoptera metacommunities by reciprocal average, we identify checkerboard patterns (negative coherence) at Guiana, Inambari, Tapajós and Xingú interfluves (Table 1). However, when the metacommunities of each interfluve were ordered according to environmental and geographical distance, a Quasi-nested pattern appears (Table 1).

**Table 1:** Metacommunity patterns within Amazonian interfluves. Coherence (C), measured as the number of uninterrupted presences, ordered along environmental gradients and also according to biogeographic and spatial predictors.

	**Interfluve**	**Covariate**	**C obs**	**Z**	**p**	**C null**	***σ*C**	***β* obs**	**Z**	**p**	***β* null**	*σβ*	**I**	**p**	**df**	**Pattern**
**Zygoptera**	**Guiana**	Space	38	−1.146	0.252	31.637	5.551	**-**	**-**	**-**	**-**	**-**	**-**	**-**	**-**	Random
		HII	42	−1.529	0.126	33.442	5.598	**-**	**-**	**-**	**-**	**-**	**-**	**-**	**-**	Random
		pH	37	−0.803	0.422	32.363	5.774	**-**	**-**	**-**	**-**	**-**	**-**	**-**	**-**	Random
		Width	36	−0.625	0.532	32.605	5.429	**-**	**-**	**-**	**-**	**-**	**-**	**-**	**-**	Random
		Depth	40	−1.101	0.271	33.176	6.197	**-**	**-**	**-**	**-**	**-**	**-**	**-**	**-**	Random
		Temperature	44	−1.849	0.064	32.781	6.067	**-**	**-**	**-**	**-**	**-**	**-**	**-**	**-**	Random
		Seasonality	44	−1.849	0.064	32.781	6.067	**-**	**-**	**-**	**-**	**-**	**-**	**-**	**-**	Random
		Precipitation	44	−1.849	0.064	32.781	6.067	**-**	**-**	**-**	**-**	**-**	**-**	**-**	**-**	Random
		EVI	39	−1.211	0.226	32.252	5.573	**-**	**-**	**-**	**-**	**-**	**-**	**-**	**-**	Random
		Recip Avg	21	2.160	**0.031**	33.047	5.578	225	−3.512	0.000	51.123	49.514	1.346	0.118	6	Checkerboard
	**Inambari**	Space	371	−5.902	**0.000**	235.101	23.024	0	1.011	0.312	854.413	844.922	1.391	**<0.001**	9	Quasi-Nested
		HII	374	−6.104	**0.000**	241.675	21.677	70	0.847	0.397	665.308	703.041	1.445	**<0.001**	9	Quasi-Nested
		pH	377	−5.978	**0.000**	240.080	22.904	0	0.811	0.417	687.668	847.776	1.483	**<0.001**	9	Quasi-Nested
		Width	358	−5.664	**0.000**	237.306	21.310	0	0.965	0.335	731.044	757.835	1.421	**<0.001**	9	Quasi-Nested
		Depth	370	−5.794	**0.000**	237.081	22.943	0	0.844	0.399	598.815	709.308	1.304	**<0.001**	9	Quasi-Nested
		Temperature	368	−5.639	**0.000**	237.731	23.101	0	0.798	0.425	730.216	914.724	1.481	**<0.001**	9	Quasi-Nested
		Seasonality	361	−4.964	**0.000**	242.247	23.925	33	0.936	0.349	720.936	735.008	1.552	**<0.001**	9	Quasi-Nested
		Precipitation	360	−5.246	**0.000**	240.332	22.813	161	0.691	0.490	680.608	751.875	1.543	**<0.001**	9	Quasi-Nested
		EVI	386	−6.108	**0.000**	242.603	23.475	0	0.820	0.412	766.901	935.077	1.347	**<0.001**	9	Quasi-Nested
		Recip Avg	156	3.251	**0.001**	231.905	23.346	7343	−6.275	0.001	1002.439	1010.508	1.507	**<0.001**	9	Checkerboard
	**Tapajós**	Space	181	−2.489	**0.013**	149.182	12.784	60	0.442	0.658	133.455	166.071	1.356	**0.051**	13	Quasi-Nested
		HII	179	−2.788	**0.005**	145.369	12.061	51	0.581	0.561	161.120	189.639	0.966	0.475	13	Quasi-Nested
		pH	177	−2.691	**0.007**	140.645	13.509	12	0.885	0.376	173.544	182.503	1.048	0.334	13	Quasi-Nested
		Width	189	−3.355	**0.001**	147.422	12.391	0	0.905	0.365	110.423	121.982	1.516	**0.015**	13	Quasi-Nested
		Depth	181	−2.586	**0.010**	145.938	13.557	60	0.517	0.605	140.837	156.384	1.140	0.178	13	Quasi-Nested
		Temperature	176	−2.415	**0.016**	143.486	13.462	1	0.939	0.348	234.420	248.662	0.988	0.523	13	Quasi-Nested
		Seasonality	176	−2.363	**0.018**	146.653	12.420	48	0.631	0.528	154.445	168.701	1.234	0.094	13	Quasi-Nested
		Precipitation	179	−2.866	**0.004**	143.742	12.300	0	0.867	0.386	151.147	174.292	1.034	0.376	13	Quasi-Nested
		EVI	178	−2.330	**0.020**	149.651	12.164	2	0.963	0.336	137.344	140.533	1.793	**0.001**	13	Quasi-Nested
		Recip Avg	102	3.790	**0.000**	149.430	12.514	1292	−5.682	0.001	160.900	199.065	1.292	0.088	13	Checkerboard
	**Xingu**	Space	145	−6.663	**<0.001**	79.760	9.791	24	1.074	0.283	384.555	335.613	1.067	0.212	7	Quasi-Nested
		HII	149	−7.241	**<0.001**	82.725	9.152	0	1.028	0.304	299.646	291.511	0.978	0.486	7	Quasi-Nested
		pH	133	−4.792	**<0.001**	81.503	10.747	74	0.931	0.352	375.743	324.208	0.949	0.360	7	Quasi-Nested
		Width	134	−5.497	**<0.001**	81.414	9.565	29	1.052	0.293	333.718	289.762	1.009	0.396	7	Quasi-Nested
		Depth	148	−7.266	**<0.001**	83.149	8.925	0	1.046	0.295	319.940	305.793	0.970	0.460	7	Quasi-Nested
		Temperature	134	−6.152	**<0.001**	80.349	8.721	112	0.774	0.439	320.683	269.462	0.957	0.369	7	Quasi-Nested
		Seasonality	135	−5.926	**<0.001**	80.001	9.281	86	0.756	0.449	348.003	346.395	0.949	0.360	7	Quasi-Nested
		Precipitation	135	−5.926	**<0.001**	80.001	9.281	86	0.756	0.449	348.003	346.395	0.949	0.360	7	Quasi-Nested
		EVI	143	−6.290	**<0.001**	82.337	9.644	0	1.168	0.243	304.040	260.386	0.964	0.413	7	Quasi-Nested
		Recip Avg	44	3.838	**<0.001**	79.590	9.273	1153	−2.596	0.009	374.887	299.681	1.016	0.373	7	Checkerboard
**Anisoptera**	**Tapajós**	Space	21	1.329	0.184	29.022	6.036	**-**	**-**	**-**	**-**	**-**	**-**	**-**	**-**	Random
		HII	19	1.440	0.150	27.478	5.889	**-**	**-**	**-**	**-**	**-**	**-**	**-**	**-**	Random
		pH	22	0.446	0.655	24.557	5.730	**-**	**-**	**-**	**-**	**-**	**-**	**-**	**-**	Random
		Width	20	1.286	0.198	28.280	6.437	**-**	**-**	**-**	**-**	**-**	**-**	**-**	**-**	Random
		Depth	19	1.726	0.084	29.514	6.091	**-**	**-**	**-**	**-**	**-**	**-**	**-**	**-**	Random
		Temperature	18	1.139	0.255	24.097	5.354	**-**	**-**	**-**	**-**	**-**	**-**	**-**	**-**	Random
		Seasonality	23	0.709	0.478	27.318	6.088	**-**	**-**	**-**	**-**	**-**	**-**	**-**	**-**	Random
		Precipitation	21	1.033	0.301	27.004	5.810	**-**	**-**	**-**	**-**	**-**	**-**	**-**	**-**	Random
		EVI	21	1.033	0.301	27.388	6.182	**-**	**-**	**-**	**-**	**-**	**-**	**-**	**-**	Random
		Recip Avg	19	0.607	0.544	22.574	5.888	**-**	**-**	**-**	**-**	**-**	**-**	**-**	**-**	Random

**Notes.**

C obs observed coherence C null coherence expected by chance, based on the 999 null metacommunities *σ*C standard deviation of coherence

Bold indicates statistically significant *p*-values.

**Figure 3:**
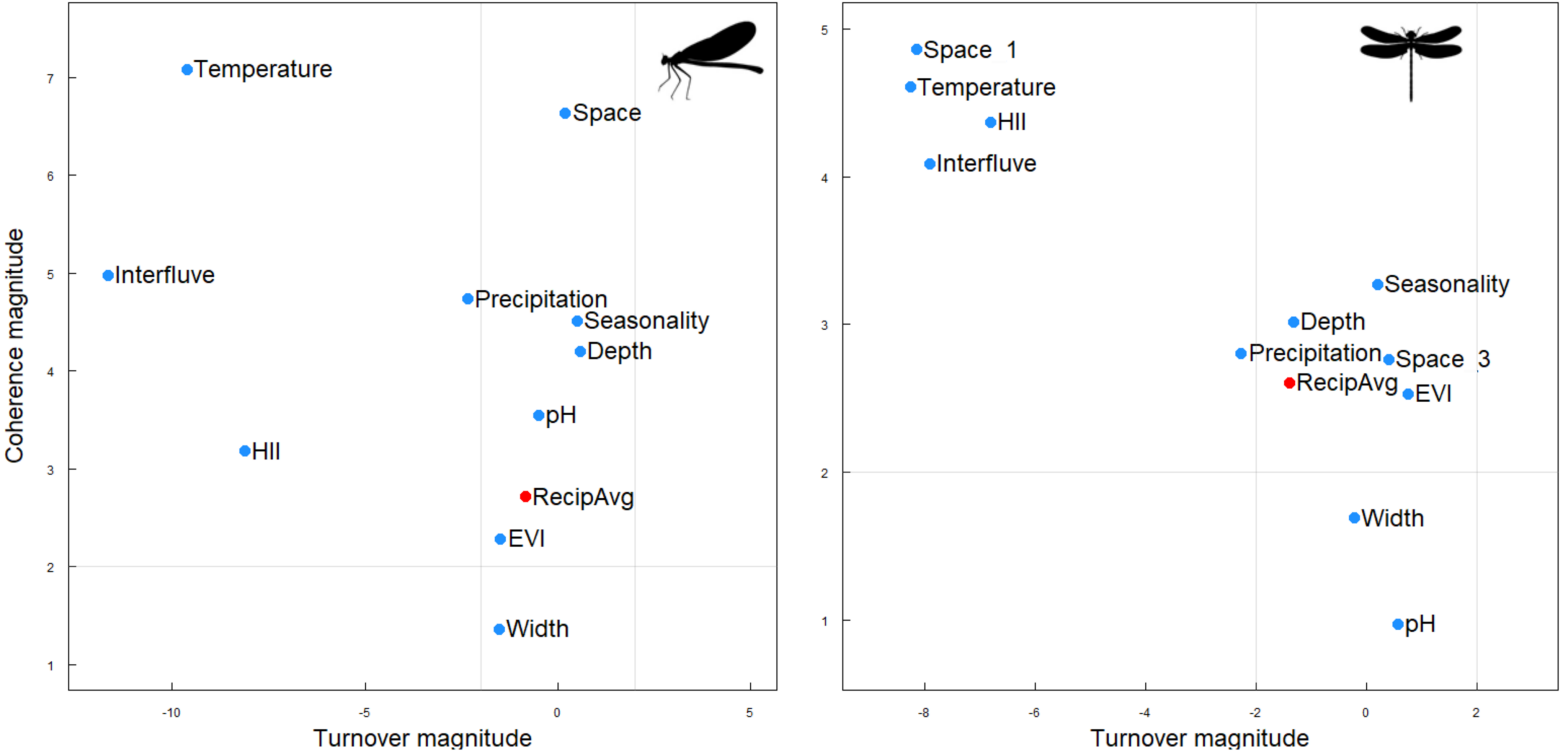
Structure of the Zygoptera (A) and Anisoptera (B) metacommunities at Amazonia. Metacommunities showed Clementsian patterns when sites were ordered by environmental variables. In red, metacommunities ordered by reciprocal averaging gradient. The magnitude of the difference (Z) between the null expectation and the empirical statistics for coherence (*y*-axis), and turnover (*x*-axis) determines the location of the metacommunity in the phase space. Gray lines separate regions based on statistical significance (*a* < 0.05) relative to a null expectation (adapted from Dallas et al. (2016)).

Contrary to our expectations, however, both Zygoptera and Anisoptera showed very similar results across interfluves. Metacommunities of both suborders showed a *Clementsian* pattern: that is, species replaced each other and their distributions were largely driven by the interfluve, temperature and habitat integrity, and additionally by geographical distance in the Anisoptera metacommunities (Fig. 3, Table 2).

**Figure 4:**
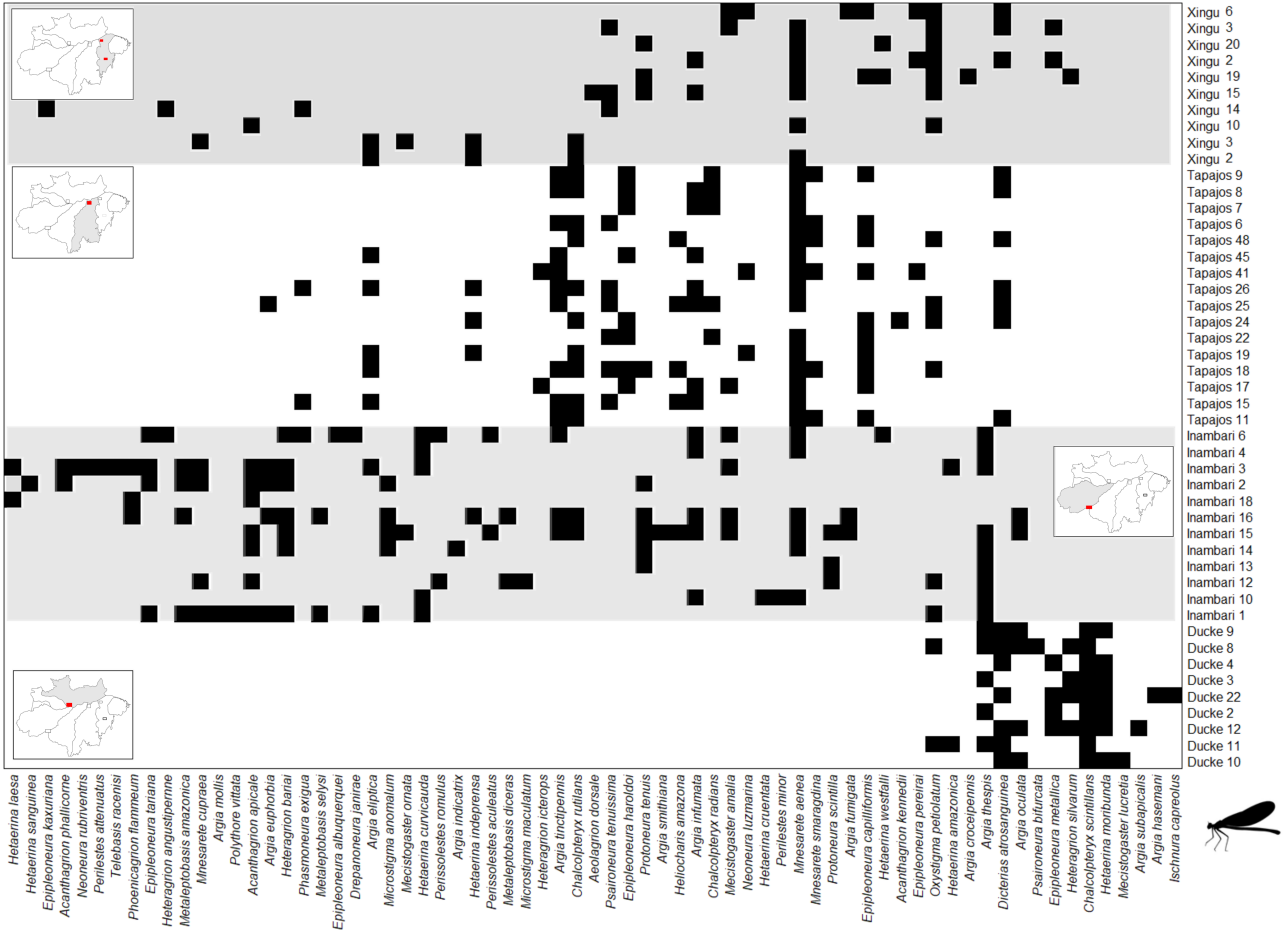
Clementsian pattern of Zygoptera metacommunities ordered by interfluve –the variable that showed the greatest magnitude of difference between observed and expected matrices (Z). Sites are depicted on rows and species on columns. Black squares indicate species occurrences; in the Clementsian pattern, particular species groups appear in different compartments.

**Figure 5:**
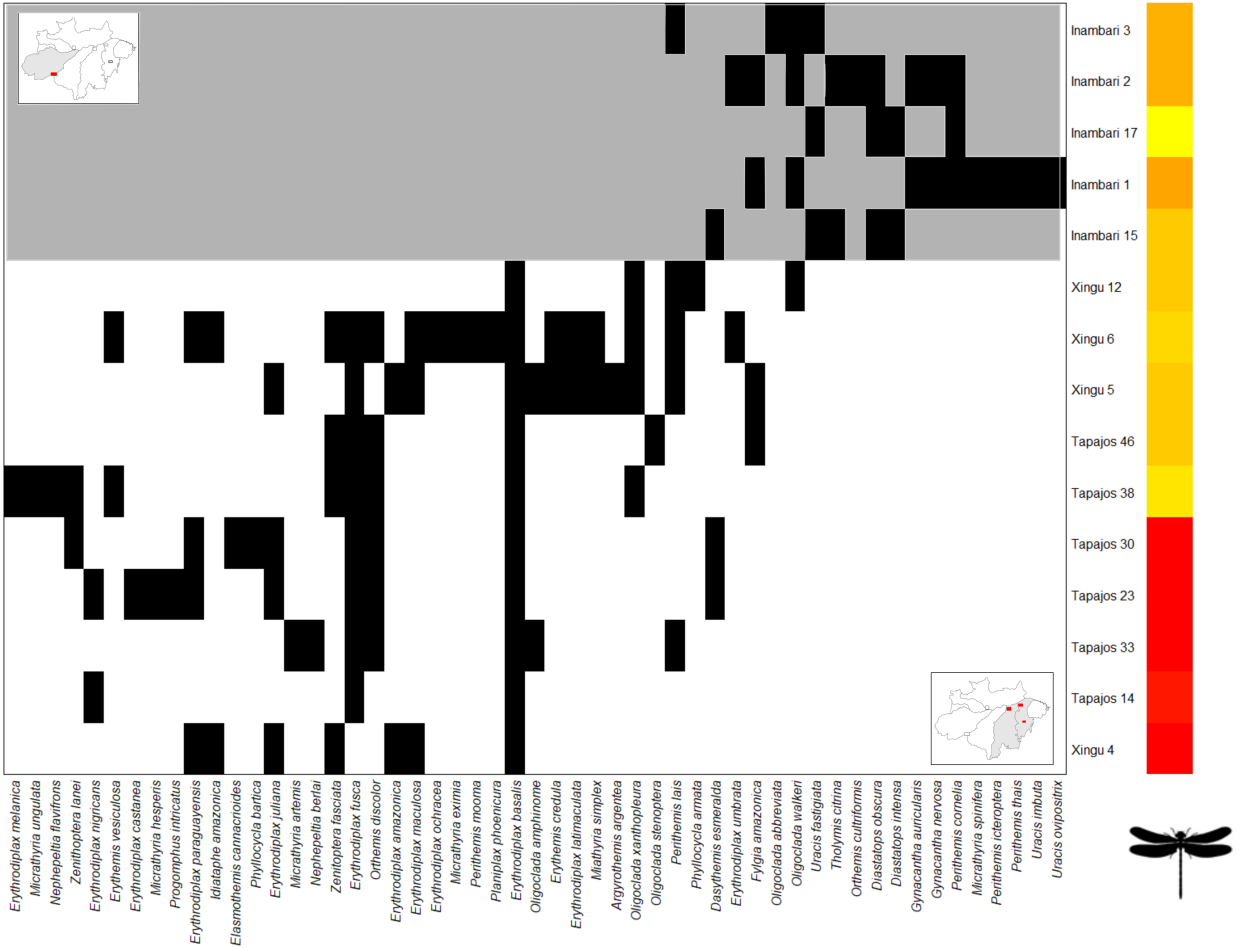
Clementsian pattern of Anisoptera metacommunities ordered by temperature –the variable that showed the greatest magnitude of difference between observed and expected matrices (Z). Sites are depicted on rows and species on columns. Black squares indicate species occurrences; in the Clementsian pattern, particular species groups appear in different compartments.

The *Clementsian* pattern observed for Zygoptera revealed that the distribution of some species of this suborder are restricted to individual interfluves. For example, *Chalcopteryx scintillans* McLachlan, 1870 and *Hetaerina moribunda* Hagen in Selys, 1853 were restricted to the Guiana interfluve, while *Acanthagrion phallicorne* Leonard, 1977 and *Hetaerina laesa* Hagen in Selys, 1853 were restricted to the Inambari interfluve (Fig. 4). Likewise, some Anisoptera species were restricted to one rather than other interfluve. Some species, like *Erythrodiplax paraguayensis* (Förster, 1905), *E. juliana* Ris, 1911, *Idiathape amazonica* (Kirby, 1889) among others, were exclusive to Xingú and Tapajós interfluves, while the species *Tholimis citrina* Hagen, 1867, *Diastatops obscura* (Fabricius, 1775), *D. intensa* Montgomery, 1940, *Gynacantha auricularis* Martin, 1909, *Gynacantha nervosa* Rambur, 1842, and *Perithemis cornelia* Ris, 1910 were exclusive to Inambari (Fig. 5).

## Discussion

Our results suggest that the spatial structure displayed by Odonata metacommunities varies with scale, showing different patterns depending on whether we look across or within interfluves. When looking across interfluves, we observed that both Zygoptera and Anisoptera present a *Clementsian* pattern, possibly driven by similar environmental factors and constrained by the location of major rivers. Indeed, annual mean temperature, habitat integrity, annual precipitation and the biogeographic region (i.e., interfluve) in which the metacommunity is placed are the main correlates of the spatial structure of Odonata metacommunities across the Amazonian interfluves. This suggests that both Zygoptera and Anisoptera metacommunities are arranged in subsets of communities along environmental gradients separated by the major Amazonian rivers (Alves-Martins et al., 2018; Brasil et al., 2017). However, these results are less consistent within interfluves, and only partially support our initial hypothesis. The spatial structure of Zygoptera metacommunities at Guiana and Anisoptera metacommunities at Tapajós interfluves were both classified as random, suggesting that neutral process may play a major role at smaller spatial scales (Meynard et al., 2013). Moreover, the spatial structure of Zygoptera metacommunities at Inambari, Tapajós and Xingu interfluves displayed varying patterns. When ordered along environmental and spatial gradients, the spatial structure of these metacommunities displayed a Quasi-nested pattern. This indicates that there is a progressive loss of species across communities throughout both spatial and environmental gradients. Yet when ordered along community ordination axes through reciprocal averaging (Leibold & Mikkelson, 2002), these metacommunities showed checkerboard patterns.

**Table 2:** Clementsian pattern in the Zygoptera and Anisoptera metacommunities in the Amazonia, by the evaluation of three EMS - Coherence, Turnover and Boundary Clumping.

	**Covariate**	**C obs**	**Z**	**p**	**C null**	***σ*C**	***β* obs**	**Z**	**p**	***β* null**	*σβ*	**I**	**p**	**df**	**Pattern**
Zygoptera	Space	1317	6.636	**<0.001**	1948.614	95.175	2116	0.195	0.845	2811.973	3563.579	1.921	**<0.001**	44	Quasi-Nested
	HII	1624	3.181	**0.001**	1913.390	90.970	51062	−8.109	**<0.001**	4144.332	5785.557	2.359	**<0.001**	44	**Clementsian**
	pH	1544	3.549	**<0.001**	1893.314	98.440	6704	−0.510	0.610	4146.172	5016.043	1.766	**<0.001**	44	Quasi-Clementsian
	Width	1784	1.364	0.173	1899.836	84.948	6452	−1.521	0.128	2748.147	2435.887	2.366	**<0.001**	44	Quasi-Clementsian
	Depth	1496	4.302	**<0.001**	1881.383	89.588	941	0.579	0.562	3117.047	3756.452	2.055	**<0.001**	44	Quasi-Nested
	Temperature	1144	7.080	**<0.002**	1819.858	95.454	108949	−9.608	**0.000**	5704.781	10745.637	1.853	**<0.001**	44	**Clementsian**
	Seasonality	1446	4.511	**<0.003**	1897.320	100.039	820	0.491	0.624	2791.771	4019.270	1.773	**<0.001**	44	Quasi-Nested
	Precipitation	1314	4.636	**<0.004**	1869.521	119.827	15140	−2.347	**0.019**	3383.697	5008.044	1.720	**<0.001**	44	**Clementsian**
	EVI	1695	2.283	**0.022**	1897.436	88.687	7576	−1.484	0.138	2968.212	3105.611	1.910	**<0.001**	44	Quasi-Clementsian
	Interfluve	1271	4.982	**<0.001**	1815.074	109.218	50081	−11.671	**<0.001**	3428.736	3997.394	2.038	**<0.001**	44	**Clementsian**
	RecipAvg	1153	2.716	**0.007**	1541.730	143.116	139041	−0.844	0.399	108109.812	36641.035	1.766	**<0.001**	44	
Anisoptera	Spatial Filter 1	213	4.636	**<0.001**	341.533	27.727	15234	−8.149	**<0.001**	1481.541	1687.534	3.238	**<0.001**	50	**Clementsian**
	Spatial Filter 3	262	2.663	**0.008**	341.483	29.842	1080	0.399	0.690	1735.172	1643.009	4.587	**<0.001**	50	Quasi-Nested
	HII	202	4.368	**<0.001**	339.207	31.409	14799	−6.804	**<0.001**	2045.374	1874.318	5.802	**<0.001**	50	**Clementsian**
	pH	305	0.974	0.330	330.443	26.109	851	0.572	0.567	1744.565	1561.156	15.111	**<0.001**	50	Quasi-Nested
	Width	288	1.691	0.091	338.812	30.053	1916	−0.217	0.829	1632.327	1309.885	7.960	**<0.001**	50	Quasi-Clementsian
	Depth	259	3.017	**0.003**	341.900	27.474	3809	−1.319	0.187	1708.445	1591.967	6.476	**<0.001**	50	Quasi-Clementsian
	Temperature	206	4.604	**<0.001**	329.883	26.908	14867	−8.254	**<0.001**	1697.220	1595.500	4.183	**<0.001**	50	**Clementsian**
	Seasonality	256	3.271	**0.001**	349.730	28.656	919	0.206	0.837	1179.262	1262.977	7.286	**<0.001**	50	Quasi-Nested
	Precipitation	256	2.803	**0.005**	336.011	28.548	5421	−2.265	**0.024**	1663.722	1658.869	5.667	**<0.001**	50	**Clementsian**
	EVI	281	2.533	**0.011**	350.561	27.459	408	0.747	0.455	1617.110	1619.153	7.825	**<0.001**	50	Quasi-Nested
	Interfluve	231	4.087	**<0.001**	352.406	29.703	13046	−7.903	**<0.001**	1250.468	1492.559	5.667	**<0.001**	50	**Clementsian**
	RecipAvg	198	2.608	**0.009**	278.034	30.692	15860	−1.391	0.164	11659.885	3019.654	3.238	**<0.001**	50	

**Notes.**

C obs observed coherence C null coherence expected by chance, based on the 999 null metacommunities *σ* C standard deviation of coherence *β* obs observed turnover *β* null turnover expected by chance, based on the 999 null metacommunities *σβ* standard deviation of turnover I Morisita index

Bold indicates statistically significant *p*-values.

However, our results also show that some Odonata metacommunities follow a random spatial pattern within the Guiana interfluve. Such pattern may be associated to neutral community dynamics at the smaller spatial scales of our analyses, at least in the case of Zygoptera at Guiana. Here it is important to take into account that, within each interfluve, most surveyed streams were close to each other (∼1.5 km). At this extent, environmental conditions may be relatively homogeneous throughout space (Maurer & McGill, 2004). As a consequence, spatial variation in community structure is a result of chance events (Sanders et al., 2007). Applying the same framework, Bried & Siepielski (2018) obtained similar results in a study on North American *Enallagma* damselflies, finding widespread species turnover at larger spatial scales (USA and larger watersheds) and random patterns in the smaller watersheds. In the case of Anisoptera metacommunities at Tapajós, it may be a result of the dispersal ability of these species (Brown et al., 2011; Heino et al., 2015). In general, most Anisoptera have high dispersal capabilities (see Hof, Brandle & Brandl, 2006; Heiser & Schmitt, 2010; May, 2013; Sacchi & Hardersen, 2013), which leads to a higher probability of homogenization in the assemblages of this subfamily, erasing any structuring gradient (Juen & Marco, 2012).

Moreover, when ordered by environmental gradients, Zygoptera metacommunities at the interfluves Inambari, Tapajós and Xingu displayed a Quasi-nested pattern. Several studies reported nested patterns for odonates (Kadoya et al., 2008; Craig, Reece & McIntyre, 2008; Bried et al., 2015) and other aquatic insect assemblages (Heino, 2011; Siqueira et al., 2012). Interestingly, one of the variables that showed the greatest magnitude of difference between observed and expected matrices (Z) in these interfluves was stream width (see Table 1). Increasing stream widths are usually associated to a decrease of forest specialists and an increase of generalist species (Paulson, 2006; De Marco, Batista & Cabette, 2015; Saito et al., 2016). Larger streams are associated to an impoverished fauna, characterized by generalist species, probably due to thermoregulatory restrictions of the forest specialists (Brasil et al., 2017; Oliveira-Junior et al., 2017), suggesting that this may be an important environmental filter for Neotropical odonates at smaller spatial scales (De Marco, Batista & Cabette, 2015).

Importantly, the contingent patterns found at the interfluve scale may be due to the problem of signal detection at low sample sizes (Zygoptera: nine communities at Guiana, 10 at Xingú, 12 at Inambari and 16 at Tapajós; Anisoptera: 5 at Inambari and 6 at Tapajós) or even due to the inaccuracy of EMS protocol in detecting metacommunity structure. The classical EMS framework has been criticized as being too simplistic and prone to identify ambiguous patterns (Ulrich & Gotelli, 2013). However, we used an extension of the EMS framework that, arguably, attenuates some of these concerns by introducing continuous measures of coherence and turnover, and ordering sites along known biological gradients instead of using only a latent gradient obtained through reciprocal averaging (Dallas et al., 2016).

The *Clementsian* pattern across interfluves in Anisoptera metacommunities is mainly caused by geographic distance, followed by temperature and major rivers, suggesting that Anisoptera metacommunity structure emerge of a combination of space, environment and biogeography of Amazonia. The influence of geographic distance reflects that metacommunities’ similarity decrease with increasing geographic distance. This patterns reflects the importance of dispersal in structuring the metacommunities (Heino, 2013), as expected for the high-dispersal Anisoptera. In Fig. 5 is possible to identify two discrete subsets of metacommunities, composed by species occurring at either the contiguous interfluves Tapajós and Xingú, or Inambari. Curiously, both subsets were mainly composed by congeneric species. For example, species of the genus *Erythrodiplax* (family Libellulidae) were predominantly collected at Tapajós and Xingú interfluves, whereas species of the genera *Gynacantha* (family Aeshnidae) and *Diastatops* species (family Libellulidae) occurred only at Inambari interfluve.

The *Clementsian* pattern in Zygoptera metacommunities across interfluves was associated with the influence of major rivers and environmental gradients. Our results suggest that there are at least three discrete Zygoptera metacommunities: one mainly formed by Guiana sites; the other formed by Inambari sites; and a third-one formed by Tapajós and Xingú sites (Fig. 4). This finding is consistent with the results of Brasil et al. (2017), who found a *Clementsian* pattern for Zygoptera metacommunities in non-impacted streams at Belém and Tapajós interfluves, and Alves-Martins et al. (2018), who found distinct species compositions across the interfluves of major Amazonian rivers. These results suggest that the *Clementsian* pattern in Amazonian Odonata may be related to the historical isolation of communities generated by the emergence of large rivers, which means that the edges of Odonata species ranges coincide with the limits of the interfluves (Juen & De Marco, 2012; Brasil et al., 2017; Alves-Martins et al., 2018). These distribution patterns follow the predictions of the theory of isolation by rivers in Amazonia, initially proposed by Wallace (1852), and that has been corroborated for other organisms (e.g., Cracraft, 1985; Bates, Haffer & Grismer, 2004).

Habitat integrity—which can be interpreted as a proxy to intactness of riparian forest (Nessimian et al., 2008)—influences the spatial structure of both Zygoptera and Anisoptera across interfluves, thereby shaping discrete metacommunities with distinct species compositions. The highest scores of habitat integrity were found in Guiana interfluve, which is covered by a hyper-diverse lowland *terra-firme* forest (De Oliveira & Mori, 1999), with a dense canopy (Juen & De Marco, 2011) that limits solar radiation at the forest floor. On the contrary, the lowest values of habitat integrity were found in Tapajós, located in a sustainably managed forest. It is covered by a lowland *terra-firme* forest, with variable canopy density due to legal logging (Schulze & Zweede, 2006), and consequently with higher solar radiation at the forest floor. We believe that the amount of solar radiation may affect Odonata compositional patterns on tropical streams by selecting species with certain thermoregulatory constraints (May, 1976; De Marco, Batista & Cabette, 2015).

Recently, De Marco et al. (2015) found that shaded streams favor colonization by small species, mainly heliotherms and thermal conformers. Their Ecophysiological Hypothesis (EH) suggests that size-related thermoregulatory constraints are the main filter for Odonata community assembly from headwaters to medium-sized tropical streams. There is an abiotic filter—temperature, mediated by forest cover—determining Odonata community assembly. The trade-off between species traits (in this case, size-related thermoregulation) and abiotic limiting factors (in this case, temperature and forest cover) lead to a segregation of species with different thermoregulatory strategies because species will predominately occur in their most suitable habitat. According to their thermoregulation strategies, Odonata species are classified as endotherms, heliotherms or thermal conformers (Heinrich, 1993; Corbet & May, 2008). Endotherms are exclusively Anisoptera species capable of warming-up by wing whirring and maintaining it by hemolymph circulation (May, 1976; Sformo & Doak, 2006). Heliotherms depend directly on the solar radiation to be active and it includes species of most representative Neotropical genus, e.g., *Erythrodiplax* (Anisoptera: Libellulidae), Hetaerina (Zygoptera: Calopterygidae) (Carvalho et al., 2013). For instance, many *Hetaerina* species (Calopterygidae) found in Tapajós, are heliotherms and usually found at spotlighted areas in the streams. Conversely, thermal conformers are exclusively Zygoptera species that present a small and delicate body and very slender abdomens, allowing a very quickly heat transfer to the environment. Species such as *Psaironeura bifurcata* (Sjöstedt, 1918) or *Heteragrion silvarum* (Sjöstedt, 1918), classified as thermal conformers (Heinrich, 1993), were mainly found in Guiana shaded streams.

The spatial structure of both Zygoptera and Anisoptera metacommunities across Amazonia also appears to be controlled by temperature gradients. For instance, a particular Zygoptera metacommunity, grouped in the Guiana interfluve, was associated with the highest values of annual mean temperature, while another metacommunity, composed by communities at the Tapajós sites, was associated with lower values of mean annual temperature. Additionally, at least two discrete Anisoptera metacommunities are associated with temperature gradient: one mainly with high temperatures at Inambari, and other with medium-to-lower temperatures at Xingú and Tapajós. Temperature exerts a widespread effect on the distribution of Odonata species (Hassall et al., 2007; Nóbrega & De Marco, 2011). The abundance of adults increases with temperature (Alves-Martins, Del-Claro & Jacobucci, 2012) because higher temperatures prolongs daily activity patterns (Corbet & May, 2008) and flight periods (Hilfert-Ruppell, 1998), and enhances flight performance (Dingemanse & Kalkman, 2008; Samejima & Tsubaki, 2010). Temperature can also affect the abundance of larvae by increasing the number of life cycles completed in a year (higher voltinism) (Corbet, 1999; Flenner, Richter & Suhling, 2010). This impact of temperature in the abundance of Odonata ultimately affects species distribution patterns (Brown, 1984) and may explain the changes in species composition across interfluves.

## Conclusions

We show that both Zygoptera and Anisoptera metacommunities present a *Clementsian* pattern across Amazonian interfluves and contingent patterns within these interfluves, suggesting that metacommunity patterns are strongly dependent on the spatial scale (Heino, 2011; Bried et al., 2015). The composition of Odonata metacommunities across interfluves in Amazonia emerges mainly from a combination of Amazonian major rivers, macroclimate and habitat integrity environment (and space, in the case of Anisoptera) in Amazonia. These findings are consistent with previous studies showing that Amazonian biogeography (Juen & Marco, 2012; Brasil et al., 2017; Alves-Martins et al., 2018) and climate are the main drivers for Odonata metacommunities at coarser spatial scales (Chovanec et al., 2015; Bried et al., 2015). Our results also confirm that intactness of riparian forest is important for Amazonian stream Odonata communities (De Marco, Batista & Cabette, 2015; De Marco et al., 2015; Miguel et al., 2017; Alves-Martins et al., 2018) and distinct Amazonian districts show distinct community composition, which has important implication for conservation. We, therefore, recommend explicitly considering the degree to which species replace each other across interfluves when setting priorities for conservation (see also Brasil et al., 2017). We also advocate for the development of new studies focused on the ecological and historical drivers of Anisoptera metacommunity in tropical systems, as these studies are now almost non-existent.

##  Supplemental Information

10.7717/peerj.6472/supp-1 Data S1 Raw data—Zygoptera and Anisoptera occurrence matrices Basin refers to the interfluves; Site is the sampled streamlets. Column names refers to species.

## Additional Information and Declarations

### Data Availability

The following information was supplied regarding data availability: Raw data can be found in the Supplemental Information.
